# Supplementary material for: Discordance of Biomarker Expression Profile between Primary Breast Cancer and Synchronous Axillary Lymph Node Metastasis in Preoperative Core Needle Biopsy
Source: Diagnostics (Basel). 2024 Jan 25;14(3):259. doi: 10.3390/diagnostics14030259 (PMC10854870; doi:10.3390/diagnostics14030259)
Supplement: Supplementary file 1 [file diagnostics-14-00259-s001.zip › diagnostics-2762671-supplementary.pdf]

Table S1. Clinical-pathological features and biomarkers expression on core needle biopsies in matched divergent primary tumors and axillary lymph node metastases of the present series. Discrepancies are outlined in bold and colored in yellow.

Case N.	Age	Size/Laterality	Histology	Nuclear grading	Necrosis	ER (%)	PR (%)	Ki67 (%)	HER2	FISH
1a*	69	3 cm/R	ILC	**2**	NA	90%	0%	8%	**0**	
1b*			ILC	**3**		90%	0%	8%	**2**	Neg.
2a*	35	1.3 cm/R	NST	**2**	Present	**5%**	0%	30%	3	
2b*			NST	**3**		**50%**	5%	NA	3	
3a*	64	1.3 cm/R	**NST micropapillary**	**2**	NA	**90%**	**90%**	25%	**2**	**Amplified**
3b1*			**ILC**	**3**		**0%**	**0%**	70%	**3**	
3b2*			**ILC**	2		**90%**	**70%**	30%	**1**	
4a*	48	2 cm/L	**NST**	2	Absent	90%	90%	40%	**1**	
4b*			**NST (different pattern, more solid)**	2		90%	90%	40%	**2**	Neg.
5a*	66	1.1 cm/L	**NST**	2	Absent	90%	60%	20%	**1**	
5b*			**NST (different pattern, cribriform)**	2		90%	60%	20%	**2**	Neg.
6a*	51	4 cm/L	**NST**	2	Absent	90%	20%	25%	**1**	
6b*			**NST micropapillary**	2		90%	20%	25%	**2**	Neg.
7a*	72	2.1 cm/R	**NST**	**2**	Absent	85%	**50%**	**15%**	**1**	
7b*			**NST (different pattern, more solid)**	**3**		85%	**4%**	**50%**	**2**	Neg.
8a*	45	2.8 cm/R	**NST/ILC**	3	NA	70%	70%	40%	**1**	
8b*			**NST**	3		70%	70%	40%	**2**	Neg.
9a*	61	2.1 cm/R	**NST/ILC**	2	NA	**8%**	0%	40%	**1**	
9b*			**NST (different pattern, more solid)**	2		**50%**	5%	40%	**2**	Neg.
10a*	67	2 cm/R	**NST/ILC**	**3**	NA	**0%**	0%	**45%**	3	
10b*			**NST**	**2**		**95%**	0%	**12%**	3	
11a1*	44	1.2 cm/R	**NST (clear cell/glicogen rich)**	3	Absent	90%	**50%**	30%	**1**	
11a2*		2.5 cm/R	**NST (clear cell/glicogen rich)**	3		90%	**5%**	20%	**2**	Neg.
11b*			**NST**	3		90%	**10%**	32%	**1**	
12a*	81	0.8 cm/R	**ILC**	3	NA	85%	0%	5%	**1**	
12b*			**ILC solid**	3		85%	0%	5%	**2**	Neg.
13a*	64	3 cm/L	**NST (different pattern)**	2	NA	90%	50%	15%	**1**	Neg.
13b*			**NST (different pattern)**	2		90%	50%	25%	**2**	
14a*	54	3 cm/R	**ILC**	2	NA	95%	90%	10%	**1**	Neg.
14b*			**ILC solid**	2		95%	90%	8%	**2**	
15a*	78	1.6 cm/R	NST/ILC	2	NA	95%	**10%**	5%	**0**	
15b*			NST			90%	**90%**	10%	**1**	
16a*	42	6 cm/L	**NST**	**2**	NA	95%	**10%**	40%	**0**	
16b*			**ILC**	**3**		95%	**80%**	35%	**2**	Neg.
17a*	60	2.5 cm/L	NST	3	NA	**10%**	0%	30%	3	
17b*			NST	3		**95%**	0%	40%	3	
18a*	52	2.3 cm/R	**Mucinous**	2	NA	95%	20%	20%	**1**	
18b*			**NST**	2		90%	1%	30%	**3**	
19a*	59	2 cm/R	**ILC/NST**	2	NA	95%	**70%**	10%	2	Neg.
19b*			**ILC**	2		95%	**10%**	20%	2	
20a*	74	5 cm/R	NST	2	NA	95%	**5%**	15%	3	
20b*			NST	2		95%	**60%**	10%	3	
21a*	73	0.5 cm/R	NST	**2**	NA	**95%**	**95%**	**7%**	**1**	
21b*			NST	**3**		**5%**	**0%**	**45%**	**3**	
22a*	70	2.6 cm/R	**ILC apocrine**[#]	**2**	NA	**95%**	**95%**	**7%**	**1**	
22b*			**NST/ILC**	**3**		**0%**	**5%**	**40%**	**3**	
23a*	60	2.5 cm/R	**NST (different pattern)**	3	NA	90%	**50%**	35%	**1**	
23b*			**NST (different pattern)**	3		90%	**0%**	25%	**2**	
24a*	46	NA/R	**NST**	2	NA	85%	70%	35%	**3**	
24b*			**NST micropapillary**	2		85%	70%	35%	**1**	
25a*	64	2 cm/R	NST	**2**	Absent	40%	20%	20%	**1**	
25b*			NST	**3**		85%	20%	30%	**2**	
26a*	57	4 cm/L	**NST/ILC (different pattern)**	2	Absent	95%	**3%**	**15%**	1	
26b*			**NST/ILC (different pattern)**	2		95%	**35%**	**40%**	1	
27a*	54	NA/R	**Mucinous**	**2**	Absent	95%	70%	**15%**	1	
27b*			**NST**	**3**		95%	55%	**45%**	1	
28a*	71	2.3 cm/R	**Metaplastic carcinoma**	3	Absent	**95%**	5%	25%	**2**	Neg.
28b*			**NST**	3		**0%**	0%	65%	**0**	
29a*	65	1.5 cm/L	NST	3	Absent	90%	10%	15%	2	**Neg.**
29b*			NST	3	**Present**	90%	10%	15%	2	**Amplified**
30a*	50	NA/R	NST	2	Present	95%	70%	40%	**2**	**Neg.**
30b*			NST	2		80%	70%	40%	**3**	

*same patient ("a": breast tumor; "b": nodal metastasis).

[#]staining positive for androgen receptors and GCDFP-15 at immunohistochemistry.

Abbreviations: ER: estrogen receptors, PR: progesterone receptors, HER2: human epidermal growth factor receptor-2, R: right, ILC: invasive lobular carcinoma, NA: not available, NST: no special type, L: left.
